# The Validity of Body Adiposity Indices in Predicting Metabolic Syndrome and Its Components among Egyptian Women

**DOI:** 10.3889/oamjms.2016.036

**Published:** 2016-03-03

**Authors:** Moushira Erfan Zaki, Sanaa Kamal, Hanaa Reyad, Walaa Yousef, Naglaa Hassan, Iman Helwa, Shams Kholoussi

**Affiliations:** 1 *Biological Anthropology Department, Medical Division, National Research Centre, Giza, Egypt*; 2 *Immunogenetics Department, Human Genetics and Genome Research Division, National Research, Centre, Giza, Egypt*

**Keywords:** Body adiposity, metabolic syndrome, women, validity

## Abstract

**AIM:**

To assess the associations between the body adiposity indices and risk of metabolic syndrome (MS) and its components in Egyptian women and to evaluate their predictive power.

**MATERIALS AND METHODS:**

This was a cross-sectional analysis performed on 180 Egyptian women aged between 25-35 years. They were 90 women with MS diagnosed by International Diabetes Federation (IDF) and 90 healthy age matched controls. Body adiposity index (BAI), body mass index (BMI), waist to hip ratio (WHR) and waist to height ratio (WHtR) were calculated and serum samples were analyzed for metabolic parameters. Receiver operating characteristic curves (ROC) was used to determine the discriminatory capacity of BAI, WHR WHtR and BMI for MS.

**RESULTS:**

Area under the curve (AUC) was highest for BIA, followed by WHR, WHtR and then BMI. All adiposity indices were significantly correlated with metabolic components and BAI had the highest correlation coefficients compared to other indices.

**CONCLUSION:**

BAI is a practical predictor for MS and has satisfactory diagnostic accuracy for diagnosing MS among Egyptian women and can be used in addition to WHR, WHtR and BMI for identifying MS in the field studies.

## Introduction

Obesity has become one of the most important public health problems. The increase in prevalence of obesity involves an increase in the prevalence of several obesity-related diseases [1–3]. Several studies show the relation between th e adipose tissue accumulation and the incidence of adverse metabolic events and, also, with a higher risk for developing metabolic diseases [4-10]. The metabolic syndrome (MS) is a set of interrelated risk factors such as hypertension, dyslipidemia, obesity and high blood glucose.

Insulin resistance together with central/abdominal or visceral obesity has been proposed as the key factors in the development of the MS. Several authors have tested the correlations between the indices of adiposity and several health outcomes [11-13]. There is no universally agreed definition for MS. Despite the use of the same index for central obesity assessment, the National Cholesterol Education Program (NCEP) Adult Treatment Panel III (ATP III) [14] and International Diabetes Federation (IDF) [15] differed in the waist circumference (WC) cut-off points. ATPIII proposed WC more than or equals 88 cm for women, whereas IDF proposed WC cut-off points based on population estimates. Body mass index (BMI), WC, and waist to hip ratio has all been tested for their relation to MS, but with no consistent results across the globe. The prevalence also varies by ethnicity. In the National Health and Nutrition Examination Survey III (NHANES III) [16], the age-adjusted prevalence was 30–40%higher in p eople of Mexican–American origin than in persons of White and African–American origin.

The highest prevalence is found in the Middle East region, where more than every third person above the age of 20 fulfils the criteria for having the metabolic syndrome. The syndrome is common and has a rising prevalence worldwide, relating largely to a complex interplay of rapid nutritional alterations, sedentary lifestyle and socioeconomic evolution, increasing affluence, rural-to urban migration, leading to obesity.

This study aims to evaluate the predictive power of adiposity indices as predictors for MS in the sample of Egyptian women.

## Material and Methods

The present cross-sectional study was carried out on 90 obese Egyptian women (aged 25-35 years old) recruited from obesity clinic National Research Centre with MS having the presence of 3 or more criteria/parameters according to the International Diabetes Federation (IDF) and 90 healthy controls without or even have a single parameter of metabolic syndrome. A written informed consent was obtained from each study subject.

IDF criteria are: central obesity (defined as waist circumference >80 cm). If BMI is > 30 kg/m^2^, central obesity can be assumed and waist circumference does not need to be measured, BMI has no relation with central obesity measured by WC, as there are some bodies have BMI > 30 Kg/m and WC less than 88 cm, triglycerides >150 mg/dL or specific treatment for this lipid abnormality, HDL-cholesterol < 50 mg/dL and blood pressure >130/85 mmHg or treatment of previously diagnosed hypertension, fasting plasma glucose >100 mg/dL or previously diagnosed type 2 diabetes. When central obesity plus two of the four previous criteria are met, a diagnosis of metabolic syndrome can be made [17]. BMI was calculated as weight (kg) divided by height (m) squared (kg/m^2^). BAI was calculated using the equation ((hip circumference)/((height) 1.5)-18) [18].

WC and hip circumference (HC) were measured using inelastic tape at the level midway between the lateral lower rib margin and iliac crest as well as at the levels of trochanters. WHR was calculated as WC divided by HC and WHtR was calculated as WC divided by height in centimeters.

### Serum analysis

Blood samples were collected after a 12-h overnight fast and stored at < 80°C until analyzed. An Olympus AU400 automatic analyzer (Olympus Corporation, Tokyo, Japan) was used to measure serum total cholesterol (TC), High Density Lipoprotein cholesterol (HDL-C), triglycerides (TG) and low Density Lipoprotein cholesterol (LDL-C) was calculated. Fasting blood glucose (FBG) was measured with commercial kits (Roche Diagnostics, Indianapolis, IN, USA) and fasting blood insulin (FBI) were determined with the Phadebas Insulin Test (Pharmacia, Uppsala, Sweden) using a radioimmunosorbent technique. The insulin sensitivity was then calculated using Homeostasis Model Assessment (HOMA-IR) according to the following formula: HOMA-IR = FBG (mmol/L) ×FBI (μU/ml)/22.5.

### Statistical Analyses

All the data were tested for their normal distribution (Kolmogorov–Smirnov test). Results are expressed as means and standard deviations (SD). Student t test for unpaired data was used to evaluate differences in anthropometric and biochemical characteristics between cases and controls.

The existence of significant bivariate correlations between parameters such as BAI, BMI, WHR, WHtR and biochemical parameters and metabolic risk factors was ascertained by determining correlation coefficients. Receiver operating characteristic curves were used to determine discriminatory capacity of BAI, WHR, WHtR and BMI for metabolic syndrome risk.

Statistical analysis was carried out using IBM SPSS Statistics 20.0 software (SPSS/IBM, Chicago, IL, USA). Significance was accepted at p<0.05.

## Results

Table 1 shows clinical, biochemical and anthropometric characteristics of the study participants. Statistically significant differences were found between the MS cases and controls in all anthropometric and biochemical parameters. Women with MS showed significant higher values of BMI, WC, HC, WHR, WHtR, BAI, blood pressure levels, LDL-C, TG, FBI, FBG, HOMA-IR and lower HDL-C than normal controls (p < 0.001).

**Table 1:** Age, anthropometric, clinical and biochemical indices characteristics of the study participants

Characteristics	MS	Non-MS
Age	28.42 ± 2.45	29.42 ± 3.56
BMI (kg/m^2^)	30.42 ± 3.70	23.51 ± 2.91 ***
Waist circumference (cm)	106.42 ± 10.00	99.46 ± 17.395 ***
Hip circumference (cm)	125.22 ± 12.89	119.01 ± 10.78 ***
WHR	0.89 ± 0.05	0.80 ± 0.13 ***
WHtR	0.72 ± 0.01	0.59 ± 0.01 ***
BAI (kg/m^2^)	35.75 ± 4.22	25.65 ± 3.31 ***
Systolic BP (mmHg	120.00 ± 12.6	115.71 ± 18.44 ***
Diastolic BP (mmHg)	94.54 ± 6.87	73.83 ± 10.43 ***
FBG (mg/dl)	112.52 ± 8. 22	90.52 ± 5.22 ***
FBI (μU/ml)	18.20 ± 0.85	8.20 ± 0.65 ***
HOMA-IR	6.88 ± 1.29	2.3 ± .99 ***
Triglycerides (mg/dl)	145.65 ± 30.61	100.41 ± 33.29 ***
Total cholesterol (mg/dl)	174.71 ± 30.81	124.71 ± 23.81 ***
LDL-C (mg/dl)	163.71 ± 35.890	112.61 ± 29.381 ***
HDL-C (mg/dl)	45.44 ± 15.217	47.86 ± 14.560 ***

BMI: body mass index; WHR: waist to hip ratio; WHtR: waist to height ratio; BAI: body adiposity Index; FBG: fasting blood glucose; FBI: fasting blood insulin; HOMA-IR: homeostasis model assessment of Insulin Resistance; HDL-C: high-density lipoprotein cholesterol; LDL-C: low-density lipoprotein cholesterol.

*** Significant at p-value < 0.001.

**Figure 1:**
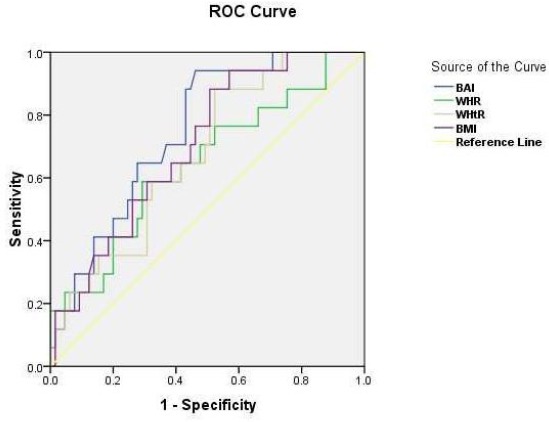
*ROC Curve to determine the diagnostic value of body adiposity indices to predict the metabolic Syndrome*.

Figure 1 shows the ROC curve for BAI, BMI and WHR with respect to the presence of MS. Area Under the Curve(AUC) was 69.8% (95% CI: 62.2% – 81. 3%) for BIA, 67.3% (95% CI:56.8% – 80.9%) for WHR,65.9% (95% CI:56.8% –79.9%)for WHtR and 61.3% (95% CI: 48.2%-78.5%) for BMI. BAI showed higher discriminatory capacity than WHR, WHtR and BMI for MS risk.

Table 2 shows the coefficients of partial correlation between anthropometric measures and metabolic risk factors. BAI had the highest correlation coefficients with metabolic components followed by WHR and WHtR and then BMI.

**Table 2:** Partial correlations between adjusted body adiposity indices and metabolic components; to exclude the effect of age

	BAI	WHR	WHtR	BMI
Systolic BP	0.727^**^	0.560^**^	0.461^**^	0.361^*^
Diastolic BP	0.686^**^	0.502^**^	0.434^**^	0.374^**^
FBG	0.586^***^	0.3214^**^	0.294^*^	0.283^*^
FBI	0.575^***^	0.402^**^	0.382^*^	0.361^*^
HOMA-IR	0.486^**^	0.482^**^	0.464^**^	0.253^*^
Triglyceride	0.485^**^	0.404^**^	0.384^*^	0.251^*^
HDL-C	-0.583^**^	-0.455^**^	-0.335^*^	-0.237^*^
LDL-C	0.486^***^	0.381^**^	0.335^*^	0.235^*^

## Discussion

The objective of this cross sectional study was to analyze correlation of body adiposity indices with metabolic risk factors among Egyptian women aged 25-35 years old. Adipose tissue accumulation increases the incidence and risk of adverse metabolic events and diseases.

To our knowledge this study is the first study focused on evaluating the applicability of BAI as a method to determine metabolic risk in a sample of Egyptian women and determine the validity of each of BAI, WHR, WHtR and BMI that might have predictive power for the risk of MS. The main finding of the present study is that BAI a good adiposity predictor for MS and overcome the limitations of BMI and the other indices analyzed. Body fat content, fat distribution or adiposity, therefore, could be considered as important indicators of metabolic risk. Body adiposity index has been developed, to overcome the shortcomings of BMI and it can be used to reflect body fat percentage (BF %) in adults [19, 20]. BAI was suggested to have several advantages over BMI, including that it yields similar associations with BF% for men and women and may be more practical to assess in field studies because it does not require a weight measurement and it can be used to reflect body fat percentage (BF %) in adults. It has been suggested that the BAI can be used to mirror %body fat for adult men and women of differing ethnicities without numerical correction.

BAI was suggested to have several advantages over BMI, including that it yields similar associations with BF% for men and women and may be more practical to assess in field studies because it does not require a weight measurement [21].

Current study tested adiposity indices for identifying MS using ROC curves and detect their sensitivity and specificity in the categorization of MS among Egyptian women.

The present results demonstrated that BAI has the higher discriminatory capacity (higher area under the curve) than the WHR, WHtR and BMI from ROC curves for identifying MS (IDF criteria). Area Under the Curve (AUC) was 69.8% for BIA, 67.3% for WHR,65.9% for WHtR and 61.3% for BMI, indicating that BMI has the weakest predictive power as compared to other adiposity indices. Moreover, partial correlation analysis showed that BAI had the highest correlation with metabolic risk factors compared to other adiposity indices. BAI level in subjects with metabolic syndrome was 35.75 ± 4.22 and 25.65 ± 3.31 in healthy controls.

The syndrome is common and has a rising prevalence worldwide, relating largely to a complex interplay of rapid nutritional alterations, sedentary lifestyle and socioeconomic evolution, increasing affluence, rural -tourban migration, leading to obesity [22].

However other studies reported that BAI could be less useful than BMI when the metabolic health risk is evaluated. Furthermore, this study suggested that WC and WHR may be even better candidates than BMI or BAI as simple (only tape measurements are required) and practical indicators of cardiovascular health risk [23].

In the IDF definition for MS, central obesity (increased WC) is a pre-requisite criterion in addition to two or more of the other major risk factors. The IDF definition was adopted in this study for identifying subjects with MS and studying the relation between different risk factors. Different measures of central obesity have been developed over time including WHR, BMI, WC, and WHtR [22-24]. The first definition of MS by World Health Organization (WHO) used BMI and WHR. The ATPIII used BMI and WC to indicate central obesity, whereas the IDF only used WC in their MS criteria.

Our study showed significant partial correlations between adiposity indices and metabolic syndrome risk factors after controlling for age with highest correlations BAI followed by WHR then WHtR and BMI. In agreement to the current study, several studies have shown that the BAI supposes a new approach in order to determine the adiposity and MS [19-24].

WHtR ratio may reflect visceral fat more accurately than WHR, since the latter indicator does not reflect visceral fat properly as it may stay the same because WC and hip circumference can increase or decrease proportionately. However, previous study from Iran demonstrated that increased WHR was a better predictor for CVD risk factors than BMI, WC and WHtR, in all age groups [25]. BAI has been suggested to have several advantages over BMI. BAI gives similar associations with BF% for men and women and may be more practical to assess in field studies because it does not require a weight measurement. Many techniques have been developed for assessing and/or determining body fat or adiposity. These include the BMI, WC, WHtR, skinfold thickness, dual energy X-ray absorption (DXA) and hydrostatic densitometry [22-26].

In our study, BMI, BAI were and WHR were significantly correlated with metabolic parameters; correlations of BAI were stronger than WHR and BMI. ROC analysis revealed also superior discrimination of BAI compared to BMI and WHR.

A recent study done in north India concluded that the correlation of BMI to percentage of body fat was better than that of BAI to percentage of body fat, the sensitivity and specificity of BAI were similar to, if not better than, BMI [27, 28].

The BAI can be measured without weighing, which renders it BAI was developed and validated in studies of Mexican American and African-American adults. Several studies of BAI values for predicting fat content or metabolic disorders in European-American, Mexican-American, Caucasian and Asian subjects have reported controversial results [29-31]. In Caucasians, BAI is a better estimate of adiposity than BMI in non-obese subjects, but less effectively than BMI in obese men and women. Another study reported that BMI more strongly correlated with BF% than BAI, and more highly associated with diabetes risk in Caucasian [32]. BMI was more accurate surrogate for adiposity in American [33, 34], Mexican Americans [35] Caucasian [36, 37] and Asian subjects [38]. Lifestyles have been changed over the preceding decades in developing countries, especially in Egypt. Increase in sedentary lifestyles has been observed that likely contribute to an increased incidence of MS [39]. Moreover, MS and its components (high waist circumference, high triglyceride levels, and low high density lipoprotein cholesterol levels) were significantly associated with menstrual irregularity in women of reproductive age [40].

In conclusion, BAI is practical predictor for MS and has a significant diagnostic accuracy for diagnosing MS among Egyptian women and can be used as a useful predictor in addition to other adiposity indices (WHR, WHtR and BMI) for identifying MS in the field studies.
